# The Architecture of Iron Microbial Mats Reflects the Adaptation of Chemolithotrophic Iron Oxidation in Freshwater and Marine Environments

**DOI:** 10.3389/fmicb.2016.00796

**Published:** 2016-06-01

**Authors:** Clara S. Chan, Sean M. McAllister, Anna H. Leavitt, Brian T. Glazer, Sean T. Krepski, David Emerson

**Affiliations:** ^1^School of Marine Science and Policy, University of DelawareNewark, DE, USA; ^2^Geological Sciences, University of DelawareNewark, DE, USA; ^3^Bigelow Laboratory for Ocean SciencesEast Boothbay, ME, USA; ^4^Department of Oceanography, University of HawaiiHonolulu, HI, USA

**Keywords:** Fe-oxidizing bacteria, microbial mats, hydrothermal vents, Zetaproteobacteria, *Leptothrix ochracea*

## Abstract

Microbes form mats with architectures that promote efficient metabolism within a particular physicochemical environment, thus studying mat structure helps us understand ecophysiology. Despite much research on chemolithotrophic Fe-oxidizing bacteria, Fe mat architecture has not been visualized because these delicate structures are easily disrupted. There are striking similarities between the biominerals that comprise freshwater and marine Fe mats, made by Beta- and Zetaproteobacteria, respectively. If these biominerals are assembled into mat structures with similar functional morphology, this would suggest that mat architecture is adapted to serve roles specific to Fe oxidation. To evaluate this, we combined light, confocal, and scanning electron microscopy of intact Fe microbial mats with experiments on sheath formation in culture, in order to understand mat developmental history and subsequently evaluate the connection between Fe oxidation and mat morphology. We sampled a freshwater sheath mat from Maine and marine stalk and sheath mats from Loihi Seamount hydrothermal vents, Hawaii. Mat morphology correlated to niche: stalks formed in steeper O_2_ gradients while sheaths were associated with low to undetectable O_2_ gradients. Fe-biomineralized filaments, twisted stalks or hollow sheaths, formed the highly porous framework of each mat. The mat-formers are keystone species, with nascent marine stalk-rich mats comprised of novel and uncommon Zetaproteobacteria. For all mats, filaments were locally highly parallel with similar morphologies, indicating that cells were synchronously tracking a chemical or physical cue. In the freshwater mat, cells inhabited sheath ends at the growing edge of the mat. Correspondingly, time lapse culture imaging showed that sheaths are made like stalks, with cells rapidly leaving behind an Fe oxide filament. The distinctive architecture common to all observed Fe mats appears to serve specific functions related to chemolithotrophic Fe oxidation, including (1) removing Fe oxyhydroxide waste without entombing cells or clogging flow paths through the mat and (2) colonizing niches where Fe(II) and O_2_ overlap. This work improves our understanding of Fe mat developmental history and how mat morphology links to metabolism. We can use these results to interpret biogenicity, metabolism, and paleoenvironmental conditions of Fe microfossil mats, which would give us insight into Earth's Fe and O_2_ history.

## Introduction

Microbial mats are intricately organized structures that provide functional advantage to their inhabitants (e.g., Stahl et al., 2013). Mat architecture reflects both ecology and physiology in that it promotes the metabolisms of the microbes that construct the mat. Chemolithotrophic microaerophilic Fe-oxidizing bacteria (FeOB) are well known for creating distinctive orange-rust colored mats where ferrous-rich fluids flow into oxygenated surface water (e.g., groundwater seeps, hydrothermal vents; Harder, 1919; Ghiorse, 1984; Emerson et al., 2010). These mats are often referred to as “flocs,” and indeed, they easily break apart and flocculate, suggesting that coherence could result from random organization. These properties make it difficult to assess if Fe mats are the product of coordinated microbial behavior, yet knowing how and why FeOB develop certain mat architectures is important to understanding FeOB habitat and ecology, as well as interpreting Fe microfossils in the rock record.

Fe microbial mats range from small, millimeters- to centimeters-thick patches where groundwater trickles from rock faces (Emerson and Revsbech, 1994) to meter-deep, 100s m^2^ fields where diffuse hydrothermal vents meet the deep sea floor (Edwards et al., 2011). In contrast to other microbial mats and biofilms, which typically include dense, tightly adherent masses of cells and extracellular polymers, Fe mats are instead composed of loosely aggregated Fe-mineralized filaments (twisted stalks, tubular sheaths) along with other morphologies. These biominerals easily disaggregate during sampling. As a result, despite much study on Fe mat community composition (e.g., McAllister et al., 2011; Baskar et al., 2012; Hegler et al., 2012; Scott et al., 2015) and mat-derived FeOB cultures (e.g., Hanert, 1973; Hallbeck et al., 1993; Emerson et al., 2007), we lack basic information about Fe mat architecture and how cells develop and inhabit the structures within natural settings.

**Figure 1:**
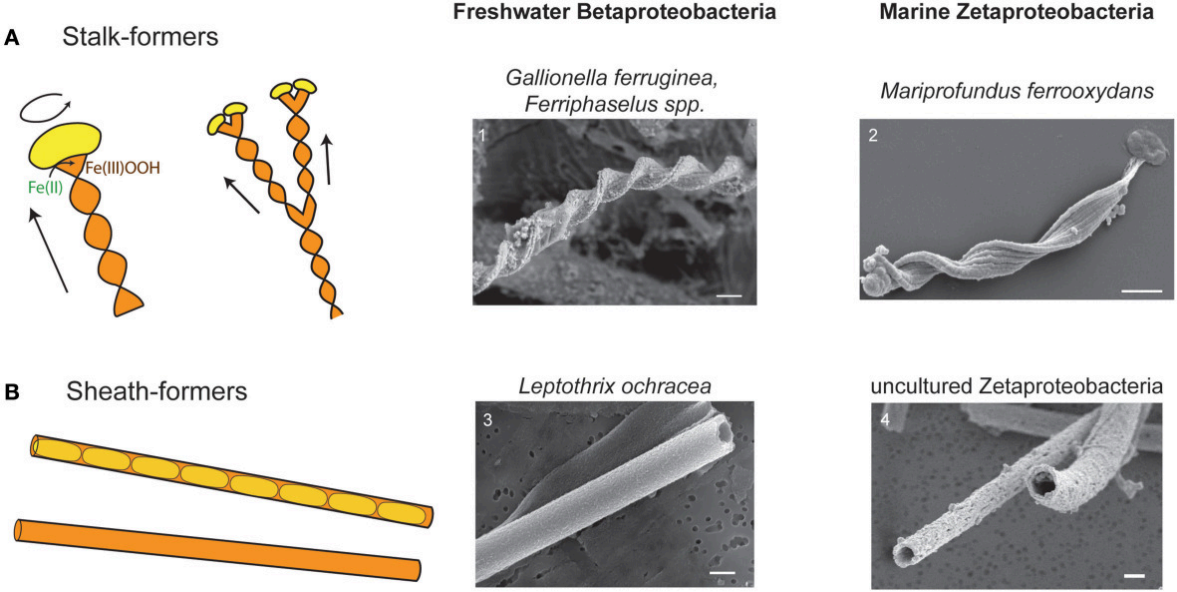
**Cartoons and SEM images of stalks and sheaths**. **(A)** Cartoon of stalk formation: as cells (yellow) produce Fe oxyhydroxide (orange), they leave behind a ribbon-like stalk. Cell rotation causes stalk twisting. As cells divide and continue to form stalks, the stalk branches. Continued cell division results in multiple branching. SEM images of (1) *Gallionella* stalk from freshwater Fe mat, Piquette Mine, Wisconsin (Banfield et al., 2001) and (2) *M. ferrooxydans* PV-1 stalk. **(B)** Cartoon of sheaths showing cells inside sheath, and empty sheath (more typically observed). SEM images of (3) *L. ochracea* from freshwater Fe mat, Lakeside Drive, Maine (Fleming et al., 2011) and (4) sheath formed by an uncultured Zetaproteobacteria from Loihi Seamount, HI (Fleming et al., 2013). All scale bars = 1 μm.

Fe microbial mats have two compelling dichotomies, phylogenetic and morphologic (Figure 1), that enable comparative study. This provides a unique opportunity that could yield insights into how mat architectures are linked to microbial Fe oxidation. The bulk of Fe microbial mats is typically comprised of either stalks or sheaths (Figure 1). Stalks are formed by two groups of chemolithotrophic FeOB: (1) Betaproteobacteria of the family Gallionellaceae, in freshwater, (*Gallionella ferruginea*—Hanert, 1973; Hallbeck and Pedersen, 1990; *Ferriphaselus spp*.—Krepski et al., 2012; Kato et al., 2014) and (2) Zetaproteobacteria of the genus *Mariprofundus* in marine environments (Emerson et al., 2007; Chan et al., 2011). Stalk-forming isolates are all obligate chemolithoautotrophic microaerophilic FeOB that typically grow at micromolar to tens μM O_2_ concentrations at the interfaces of steep Fe(II) and O_2_ gradients (Emerson and Revsbech, 1994; Druschel et al., 2008; Glazer and Rouxel, 2009; Krepski et al., 2013; Fleming et al., 2014). Sheaths are formed either by (1) Betaproteobacteria *Leptothrix ochracea*, in freshwater (Harder, 1919; van Veen et al., 1978; Emerson and Revsbech, 1994; Fleming et al., 2014), or (2) Zetaproteobacteria in marine environments (Fleming et al., 2013). Sheath-formers tend to be found associated with higher O_2_ concentrations (Fleming et al., 2014), though these have not been well constrained in the exact areas of active sheath growth. Since sheath-formers have not been isolated, the details of Fe and C metabolism are not certain. Nonetheless, the strong correlation between these organisms and relatively high Fe(II)-environments, as well as their copious production of Fe(III) oxyhydroxides strongly suggests that sheath-forming organisms oxidize Fe(II) as a key part of their metabolism. The striking parallel between terrestrial Betaproteobacteria and marine Zetaproteobacteria suggests that the filament-based mat structure plays specific roles in promoting microbial Fe oxidation. This would prove true if all types of filament-forming FeOB use their filaments to produce mats with similar architectures, despite the fact that stalk and sheath morphologies represent different O_2_ niches, distinct cell-mineral spatial relationships (Figure 1), and likely distinct genetic mechanisms. Comparative study of the various Fe microbial mat types combined with niche characterization would help resolve whether metabolism or other factors most influence Fe mat architecture.

To understand Fe mat development, we must first understand how the individual biomineral filaments are formed. The stalk-formers are much better studied, largely because they have proven cultivable while the sheath-formers tend not to grow after a few transfers. For stalk-forming organisms, as a cell oxidizes Fe(II), it continually produces a rigid Fe(III) oxyhydroxide stalk with the cells always at the leading edge (Figure 1A; Hanert, 1973; Chan et al., 2011; Krepski et al., 2013). Thus, the stalk is a record of where cells were present and oxidizing Fe, making it possible to follow stalk morphology to determine a detailed history of mat development. Imaging of sheaths from freshwater has shown us that cells live as relatively short filaments within the sheaths, while producing copious amounts of sheath material; the end result being the sheaths are largely empty (Figure 1B; e.g., Mulder and van Veen, 1963; Emerson and Revsbech, 1994). However, detailed *in situ* analysis of sheath formation has not yet been performed. Understanding how individual sheaths form would allow us to visualize how sheath mats develop, which would then enable a more informed comparison of stalk- and sheath-based Fe mat architecture.

Thus, in this study, we combine observations of intact Fe microbial mats, both stalk and sheath-rich, with experiments on sheath formation in culture, in order to understand mat developmental history and subsequently evaluate the connection between Fe oxidation and mat morphology. We sampled mats from a representative freshwater system (Spruce Point Creek, Maine; sheath-rich) and deep sea hydrothermal vents (Loihi Seamount, Hawaii; stalk- and sheath-rich). We developed a new approach to sampling Fe mats in order to preserve the delicate structure, and analyzed the mats using light, confocal, and electron microscopy. Microscope chamber cultures of freshwater sheath-forming organisms allowed us to observe Fe oxidation and biomineralized sheath formation. Time lapse imaging showed that sheaths are made much like stalks are, with the cells growing at the leading edge, suggesting that both filaments serve common functions. All of the environmental Fe mats showed similarities in directionality and texture, suggesting that they have a similar developmental history. Coupled to field observations and geochemical measurements, we show that Fe mat structure appears to be a function of environmental niche and the distinct challenges of microbial Fe oxidation, including efficient Fe biomineralization without encrustation and maintaining position in Fe(II) and O_2_ gradients.

## Methods

### Overview of field sites and analyses

Table 1 shows a summary of mat types sampled, with corresponding field sites and analyses. Descriptions of relevant site characteristics are included in the Results section.

**Table 1:** **Summary of samples and analyses**.

**Mat type**	**Sample sites**	**Geochemical analyses**	**Microscopy analyses**	**Molecular analyses**
Marine, stalk-rich mat (“curd”)	Loihi Seamount	*in situ* voltammetry for Fe(II) and O_2_	Light, confocal, SEM	Pyrosequencing of SSU rRNA V1-V3
Marine, sheath-rich mat (“veil”)	Loihi Seamount	*in situ* voltammetry for Fe(II) and O_2_	Light, confocal, SEM	Pyrosequencing of SSU rRNA V1-V3
Freshwater, sheath-rich mat	Spruce Point, Maine	*in situ* O_2_ optode; discrete sampling for Fe(II) by ferrozine	Light, confocal	
Freshwater, sheath-rich mat	Lakeside Drive, Maine		Time lapse light	
Freshwater, sheath-rich mat	Christina Creek, Delaware		Time lapse light	

### Marine mat observations and geochemical measurements

Fe microbial mats were observed and collected during a cruise to the Loihi Seamount, Hawaii in March 2013, on the R/V Thompson, using the remotely operated vehicle (ROV) Jason II. *In situ* Fe(II) and O_2_ analyses were performed using an *in situ* electrochemical analyzer (ISEA-III, Analytical Instrument Systems, Inc., Flemington, NJ, USA) connected to the ROV Jason-II similar to previously deployments (e.g., Luther et al., 2008; Glazer and Rouxel, 2009). Solid-state Au/Hg working electrodes, Ag/AgCl reference electrodes, and Pt counter electrodes were constructed from durable 3 mm diameter PEEK tubing and epoxy, and calibrated using standard electrochemical calibration methods (e.g., Brendel and Luther, 1995; Glazer et al., 2004; Luther et al., 2008). Parameters for individual voltammetric scans were: initial conditioning steps of −0.9 V for 5 s and −0.1 V for 2 s, then performing cyclic voltammeter by scanning from −0.1 to −1.85 V and back to −0.1 V at a scan rate of 500–2000 mV s^−1^. Data were analyzed using a combination of the manufacturer's software (Advanced Analysis, AIS, Inc.), open-source voltammetric analysis software (Voltint; Bristow and Taillefert, 2008), and a custom auto-analysis package (Glazer, unpublished).

### Marine mat sampling

For sampling, we targeted well-studied Fe-rich vent locations, where we have previously sampled mats with stalks and sheaths (e.g., Emerson and Moyer, 2002; McAllister et al., 2011; Fleming et al., 2013). Mats were sampled by ROV using either a suction sampler (where there was some cohesion) or a “scoop” sampler, which consisted of a 3″ plastic tube capped at both ends, one end with a ball valve. When handling the more delicate mats sampled with the scoop, samplers were opened in a bucket of seawater to minimize disturbance. The mat samples were eased out of the scoop sampler and collected in a sterile bowl. Subsamples for DNA analysis were frozen at –80°C. Intact pieces (~a few cm^3^) were carefully dissected with a scalpel to observe the internal structure and architecture. Dissected pieces were ~0.5–2 cm in length by ~0.5 cm in width and thickness. Samples for scanning electron microscopy (SEM) were fixed with 2.5% glutaraldehyde and washed with phosphate buffered saline (PBS). Samples for light microscopy were embedded in 0.5% high melt agarose, which was allowed to solidify at room temperature. Microscopy samples were stored at 4°C until further analysis.

### Freshwater mat sampling and measurements

At Spruce Point, Fe(II) profiles were obtained by taking discrete samples with a pipettor at 1 cm depth and horizontal intervals of 0.5 cm across the mat-water interface, and analyzing by the ferrozine method (Stookey, 1970). Oxygen concentrations were measured using a Pyroscience (Aachen, Germany) Firesting optical oxygen probe, as described by Emerson et al. (2015). The intact mats were sampled by gently scooping relatively cohesive mats directly into a 5 cm petri plate. Overlying water was removed and warm liquid 0.5% high melt agarose was added. These plates were placed on ice to solidify, which took 3–4 min, and then returned to the lab where they were fixed with gluteraldehyde within a few hours, then stored at 4°C until sectioning.

### Sectioning and staining of intact mat

Agarose-embedded samples were trimmed and sectioned to 100– 250 μm thickness using a Leica VT 1000 vibratome, then stored at 4°C until imaged. Sections were stained to visualize iron oxides and cells, using the method modified from Krepski et al. (2013). Briefly, slices were stained with a rhodamine-labeled soybean agglutinin (SBA) lectin or Ricinus Communis Agglutinin (RCA) I (Vector Laboratories, Burlingame, CA, USA) by pipetting 1 μL of lectin per 100 μL of sample directly onto the agarose slice. One mL of deionized water was added to submerge the slice, and the sample was incubated in the dark at room temperature for a minimum of 2 h with gentle agitation every 15 min. The slice was washed twice in 1 mL of deionized water for 30 min each. To stain cells, 10 μL of nucleic acid dye Syto13 (Invitrogen) per 100ul of sample was added directly to the slice. Stained slices were then used immediately for imaging.

### Light and confocal imaging

Mat slices were mounted on a microscope slide or tissue culture plate and imaged by brightfield and confocal fluorescence microscopy, either using a Zeiss Laser Scanning Microscope (LSM) 700 confocal scanning laser system coupled with a Zeiss Axiophot inverted microscope, or a Zeiss LSM 880-NLO with ZEN software (Zeiss, Jena, Germany). Slices were initially imaged by light microscopy to gain an understanding of general mat architecture, and photomosaics were obtained. On the LSM700, fluorescence imaging was performed using laser excitation at 488 and 555 nm, with a Plan-Neofluar 10 × 0.3 NA and C-Apochromat 40x (NA 1.2) water immersion objective, collecting the emitted fluorescence between 300–550 and 578–800 nm, respectively. On the LSM880, a 561 nm laser was used with a C-Apochromat 40x (NA 1.2) water immersion objective, with a 570–695 nm band pass filter.

### Brightfield light and confocal image processing and analysis

Brightfield mosaic images were stitched using the mosaicJ plugin for ImageJ. Confocal mosaic images were stitched in real time on the confocal microscope using ZEN 2. To display 3D information in 2D images, Z-stacks were merged using maximum intensity projection in ZEN 2011 (**Figures 4**, **5B–D**, **6C,E**, and **8B,D,E**). To highlight features, color-coded projection in ZEN 2011 was used to merge a Z-stack into a single image with color based on z-depth (**Figure 5A**, Supplemental Figure 2A). Other images were analyzed using the Fiji implementation of ImageJ (Schindelin et al., 2012). The number of cells was counted using the Analyze Particles function in ImageJ using thresholded maximum intensity projections of regions 2.42 to 9.10 × 10^−5^ cm^3^. Directionality measurements were taken using the ImageJ 2.0.0 OrientationJ Measure plugin (Supplemental Figures 4–6). Orientation color surveys were created using this same plugin, with Finite Difference Hessian structure tensor approximation and default settings (**Figure 6D**). Manual filament coloring was performed in Adobe Photoshop (**Figures 4**, **5B**). 3D rendering of confocal images was done using Amira 5.6 (FEI, Hillsboro, Oregon, USA) (Video 3).

### SEM sample preparation and imaging

In the laboratory, samples were re-fixed with 2.5% glutaraldehyde and 1% OsO_4_ for 1 h each in succession, with 3 washes with filtered deionized water after each step. Samples were dehydrated by a series of ethanol solutions: 10 min 25% EtOH, 20 min 50% EtOH, 30 min 75% EtOH, 30 min 95% EtOH, 2 × 30 min 100% EtOH. Samples were then critical point dried (Tousimis Autosamdri-815B, Rockville, MD), after which they were dissected and sputter coated with Au-Pd (Leica EM ACE600, Wetzlar, Germany). Samples were imaged in a Hitachi S-4700 field emission SEM equipped with a Oxford Instruments INCAx-act energy dispersive X-ray spectrometer. SEM images were imported into Adobe Photoshop for brightness and contrast adjustment.

### Microslide culturing

Enrichment cultures of *Leptothrix*-like organisms were grown in Fe(II)/O_2_ gradients in either flat glass capillaries (Vitrocom, Mountain Lakes, NJ) or microscope growth chambers constructed from glass slides and coverslips, as described by Krepski et al. (2013). The inoculum was a sheath-rich freshwater mat sample collected from one of several sites (Christina Creek, Delaware (Krepski et al., 2012); White Clay Creek, Delaware; or Lakeside Drive stream, Maine (Fleming et al., 2014). Filtered environmental water was used as medium, and an opposing Fe(II)/O_2_ gradient was constructed by adding FeS to one end and leaving the other end open to air. Still, mosaic, and time lapse imaging was performed either on an Olympus BX60 or Zeiss Axioimager microscope with Zeiss Axiovision.

### DNA extraction, sequencing, and analysis

DNA was extracted from two marine stalk-rich samples using the MoBio PowerSoil DNA Isolation kit. Tagged pyrosequencing of the V1-V3 region of the SSU rRNA gene was performed at the Research and Testing Laboratory (Lubbock, TX, USA). Sequences were classified using the SILVAngs pipeline against the SILVA reference database release 123 (Quast et al., 2013). Data were deposited at NCBI as short read archive accession number SRP072437. To avoid bias from microbes introduced during intact mat processing, we only analyzed and deposited Zetaproteobacterial sequences (876/4332, or 20% of high quality reads in Sample 1; 2777/7477, or 37% of Sample 2). Representatives from each OTU called by the SILVAngs pipeline were aligned against the Arb-SILVA database using the SINA Webaligner (Pruesse et al., 2012), added to a database of Zetaproteobacteria sequences, masked to 320 bp, and binned into OTUs using DOTUR (Schloss and Handelsman, 2005) to assign OTUs at 97% identity based on a curated alignment. Sequences were either assigned to known Zetaproteobacteria OTUs from McAllister et al. (2011) or considered new OTUs. Maximum likelihood phylogeny of our sequences was determined using RAxML 7.2.6 using the general time reversible (GTR) model with GAMMA approximation and 1000 bootstraps (Stamatakis, 2006).

## Results

Our previous work with natural Fe microbial mats taught us that the mats typically disaggregate under any significant shear force. Therefore, we developed a sampling and processing strategy in which we gently dislodge the mat from the substrate, minimizing disturbance during sample handling. Below, we describe the microstructure of two marine mats and one freshwater mat, in the context of their physical and chemical environments. Direct access to sheath-rich freshwater mats at Spruce Point allowed rapid fixation that maintained cells in their *in situ* positions. In combination with culturing in an Fe(II)/O_2_ gradient microslide, we were able to show how sheathed FeOB make mats, which set the stage for a comparative analysis of stalk- and sheath-rich Fe mats.

### Marine stalk-rich Fe microbial mat (curd-type), loihi seamount, hawaii

**Figure 2:**
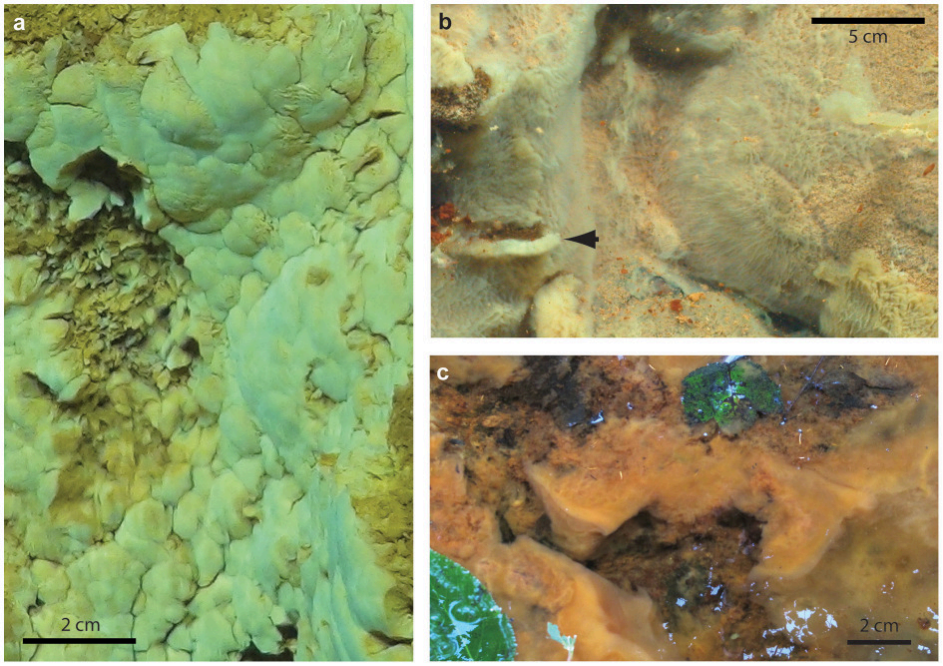
**Field pictures of Fe microbial mats**. **(A)** Loihi marine curd mat (darker area on left side is an area where sample was removed), **(B)** Loihi marine veil mat; arrow denotes dislodged mat, showing thickness, **(C)** Spruce Point freshwater mat.

The Fe-rich hydrothermal vents of the Loihi seamount host a range of Fe microbial mat types. Botryoidal mats are common on rock faces in close proximity to vent orifices (Figure 2A). Fe(II)-rich fluids flow diffusely through the mats and quickly across the face of the mats, with turbulent mixing entraining oxygen from surrounding waters. Here, aerobic microbial Fe oxidation corresponds to steep Fe(II)/O_2_ gradients, meeting at the oxic/anoxic interface at or near the surface of the mat (Table 2).

**Table 2:** **Oxygen concentration gradients in Loihi Fe mats**.

Mat type, (location^*^)	**[O_2_] at/near mat surface[μM]**	[O_2_] below^**^ mat surface[μM]	**Fe(II) at/near mat surface[μM]**	Fe(II) below^**^ mat surface[μM]
Stalk-rich curd mat (Marker 38)	99.3	79.6	<10	<10
Stalk-rich curd mat (Marker 34)	32.7	<3	309	481
Stalk-rich curd mat (Marker 34)	24.6	10.5	<10	30.0
Sheath-rich veil mat (Marker 34)	37.7	33.1	<10	<10

* *Marker names correspond to sites described by Glazer and Rouxel (2009)*.

** *For stalk-rich mats, measurements below mat surface were taken at ~7 cm depth. For sheath-rich mats, the measurement below the mat surface was taken at 1 cm depth, since the mats are much thinner*.

These mats came apart in discrete chunks, and therefore are referred to as “curds.” The curd-like mats formed in the path of venting fluid were smoother than the surrounding mats, and lighter in color, indicating they are less mineralized, which is typical of fresh mats. They easily disintegrate upon sampling; therefore a cylindrical (“scoop”) sampler was used to gently dislodge and sample intact curds as shown in Video 1.

To get insight into the populations associated with newly-forming mats, we sequenced the small subunit (SSU) ribosomal RNA genes from subsamples of the intact curd mats and found that the Zetaproteobacteria mainly consisted of four operational taxonomic units (OTUs). Following Zetaproteobacterial OTU (ZOTU) designations outlined by McAllister et al. (2011), we found the normally rare ZOTU6 dominating the Zetaproteobacterial community in one intact curd sample (434 of 857, or 50.6% of Zetaproteobacterial sequences), while another sample was dominated by three novel ZOTUs, designated here as new ZOTU N1, N2, and N3 (combined, 1900 of 2735, or 69.5% of Zetaproteobacterial sequences) (Supplemental Figure 1).

**Figure 3:**
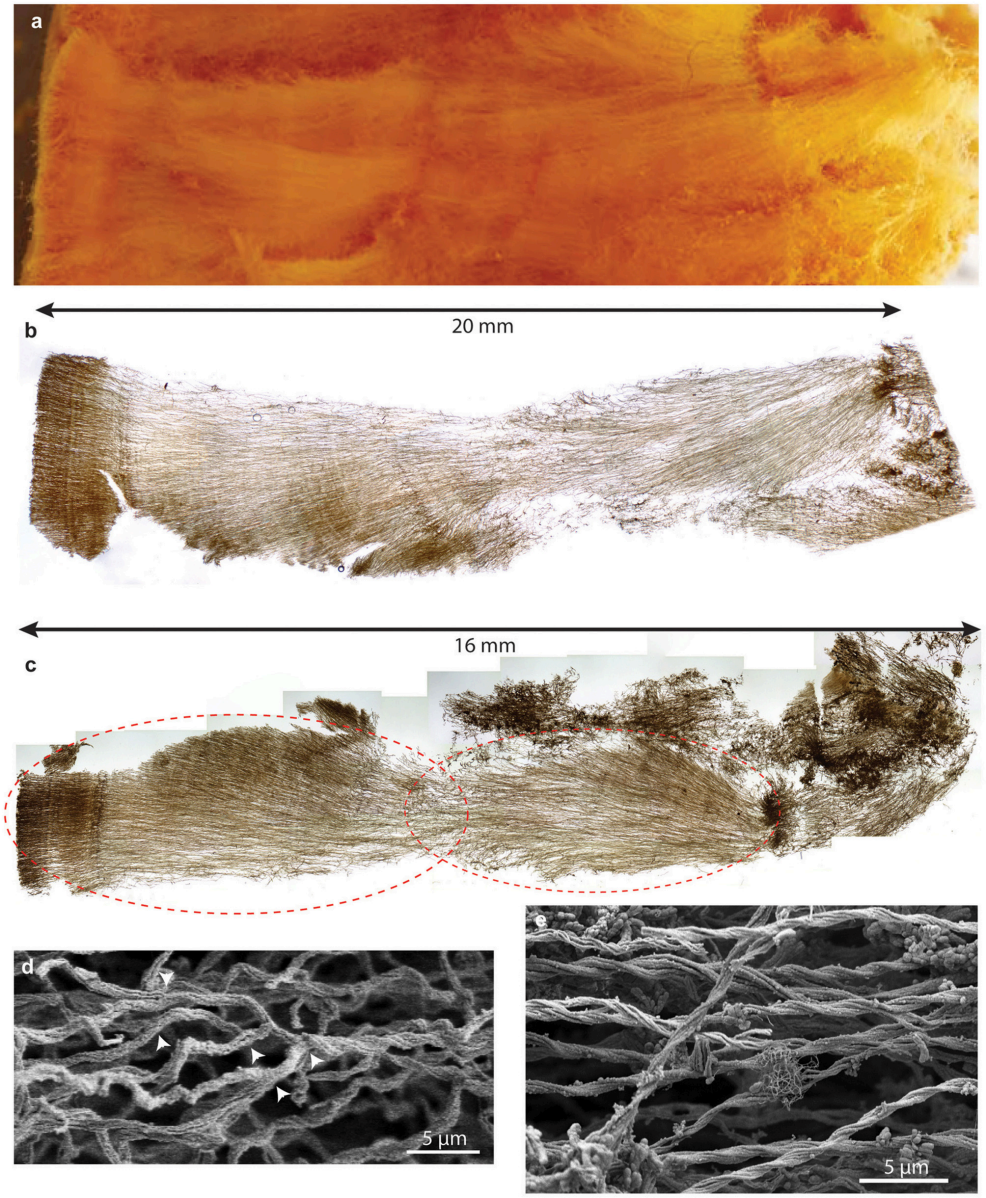
**Loihi stalk-rich curd mat**. **(A)** Macroscopic view showing linear structure. **(B)** Thin section of mat similar to that shown in **(A)**; same scale. **(C)** Thin section of another curd mat showing curd structure as radiating filamentous sections. **(D,E)** SEM images showing multiple branch points **(D)**, which result in radiation. **(E)** parallel, uniform width stalks all twisting in the same direction, suggesting coordinated growth and biomineralization.

**Figure 4:**
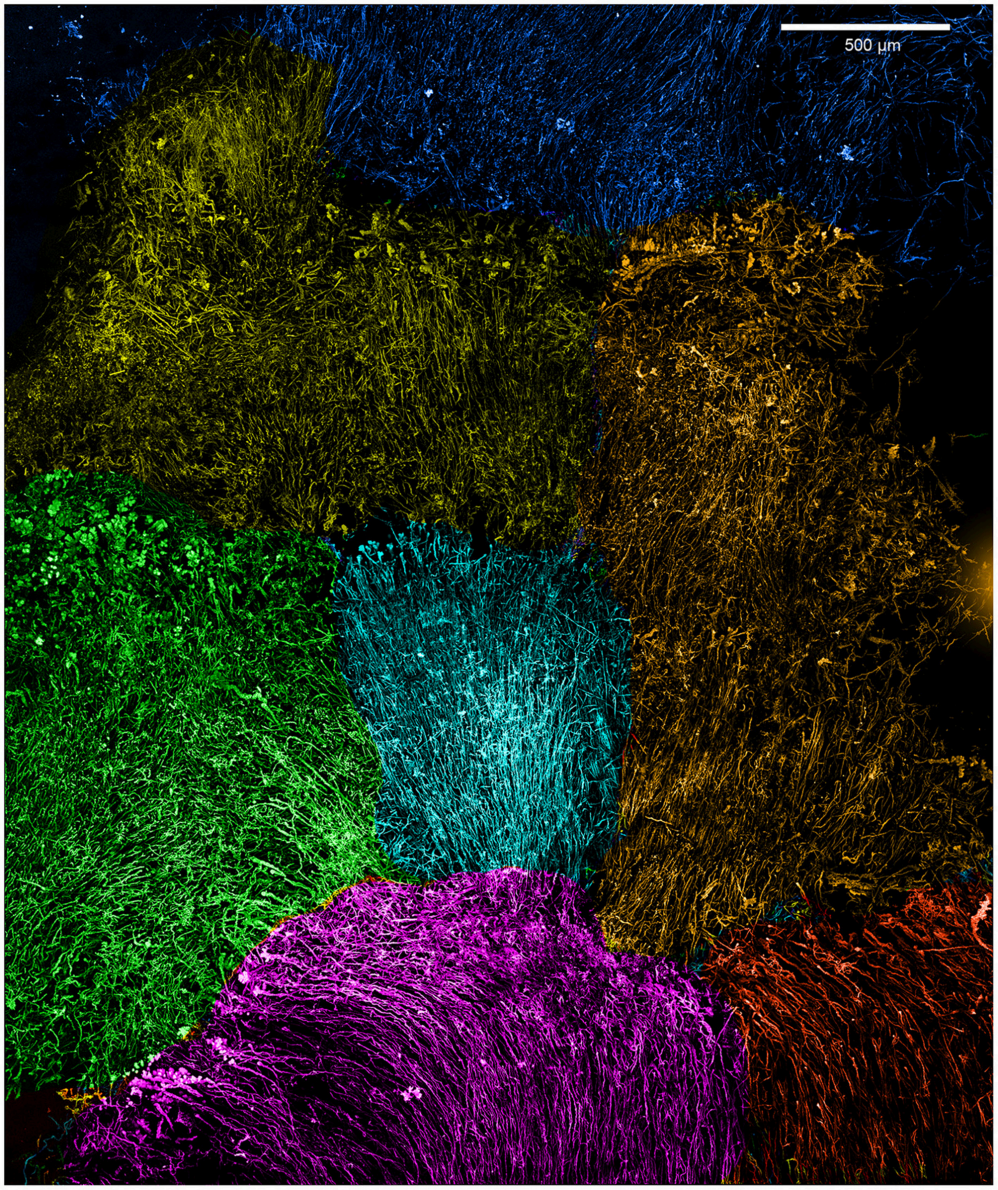
**Loihi stalk-rich curd mat confocal image, colored to highlight individual curds**.

**Figure 5:**
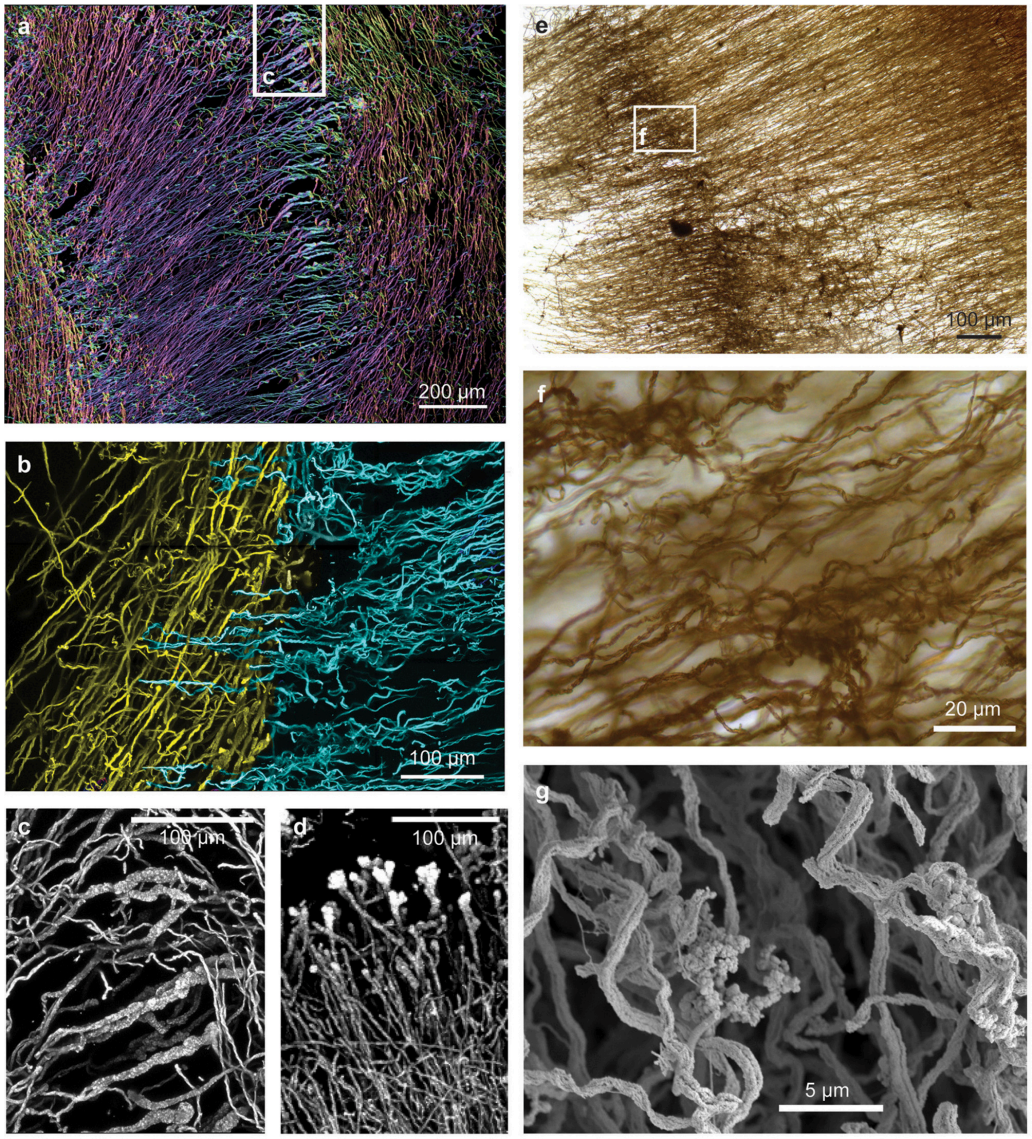
**Confocal and light microscopy images of Loihi curd mat stalks**. **(A)** Changes in stalk directions define curd boundaries. Some stalks thicken before they terminate. This image is colorized based on depth within the sample slice. **(B)** Interfaces between areas of different stalk directions; Horizontal (blue) stalks have colonized vertical (yellow) stalks. **(C,D)** Stalks that thicken at the terminal ends. **(E–G)** Mat horizon, or band, in which stalks lose their strong directionality, and instead bend and coil. See Supplemental Figures 4–6 for more images and measurements of directionality.

Initial dissection revealed that the curd interior had a filamentous texture (Figure 3A). Microscopy revealed that an individual curd is composed of highly parallel stalks that all twisted in the same direction (Figures 3B–E). Continual branching produces more stalks, causing the radiation that results in the rounded shape of each curd (Figures 2A, 3C, 4). Many stalks end at the boundaries between curds (Figures 5A–C). Using the branching patterns (e.g., Figure 3D), we were able to determine the direction of stalk growth, and therefore confirm that stalks grow toward the oxic exterior. Although we did not observe cells on the stalks, we deduce that they must have once been present at the stalk ends, at the leading edge of the mat. Many stalk ends thicken significantly just before termination (Figures 5A,C,D) or before thinning again (Supplemental Figure 2). We interpret this to be due to relatively rapid abiotic mineralization, since the stalks become featureless instead of showing the typical fibrillar structure observed with actively growing cells (Chan et al., 2011). This suggests that the environment temporarily became unfavorable, e.g., conditions that promote abiotic Fe oxidation, such as an increase in O_2_ and/or Fe(II). These results demonstrate that an interplay of biotic and abiotic Fe oxidation contribute to overall mat structure.

While most of the stalks exhibit strong directionality, there are some exceptions (Supplemental Figures 3, 5, 6). At certain horizons of the curd mat, many or all stalks change direction, turning or coiling, overall exhibiting more random orientation (Figures 5E–G; see detailed documentation in Supplemental Figures 4–6). Such variation in stalk orientations has been observed in early stages of *M. ferrooxydans* culture, before strong gradients of Fe(II) and O_2_ develop. These stalks likely record changes in some environmental cue; they may also contribute to the physical cohesiveness of the mat by acting essentially as a mesh that strengthens the mat fabric.

### Marine sheath-rich Fe microbial mat (veil-type), loihi seamount, hawaii

Although many Loihi mat samples are stalk-rich, sheaths have been observed as a minor component of bulk samples (Emerson and Moyer, 2002). In 2009, with improved lights and cameras aboard ROV Jason, we observed light orange-colored mat that appeared as a thin coating on other, darker orange microbial mats (Figure 2B). By sampling this approximately centimeter-thick surface layer, henceforth referred to as a “veil,” we retrieved a mat that was dominated by sheaths. These sheaths bore a striking resemblance to *L. ochracea*; however, Fleming et al. (2013) demonstrated that these marine sheath-formers were Zetaproteobacteria, and not related to *L. ochracea*.

On previous cruises and in this study, we observed that veils typically form further away from focused vent flow, relative to the stalk-rich curd Fe mats. Away from vents, there is little to no visible flow, and correspondingly, shallow or undetectable chemical gradients. An O_2_ concentration profile in a sheath-rich veil mat showed 38 μM O_2_ at the surface, and 33 μM O_2_ just below the surface. These mats appear fluffy and are typically thin (< 2 cm thick), making it especially challenging to obtain discrete, intact mats from the seafloor. We sampled these mats either by using a suction sampler to carefully peel them away from their substrate, or by gently loosening the mat and then allowing it to fall into a tube (“scoop”) sampler (Video 2).

**Figure 6:**
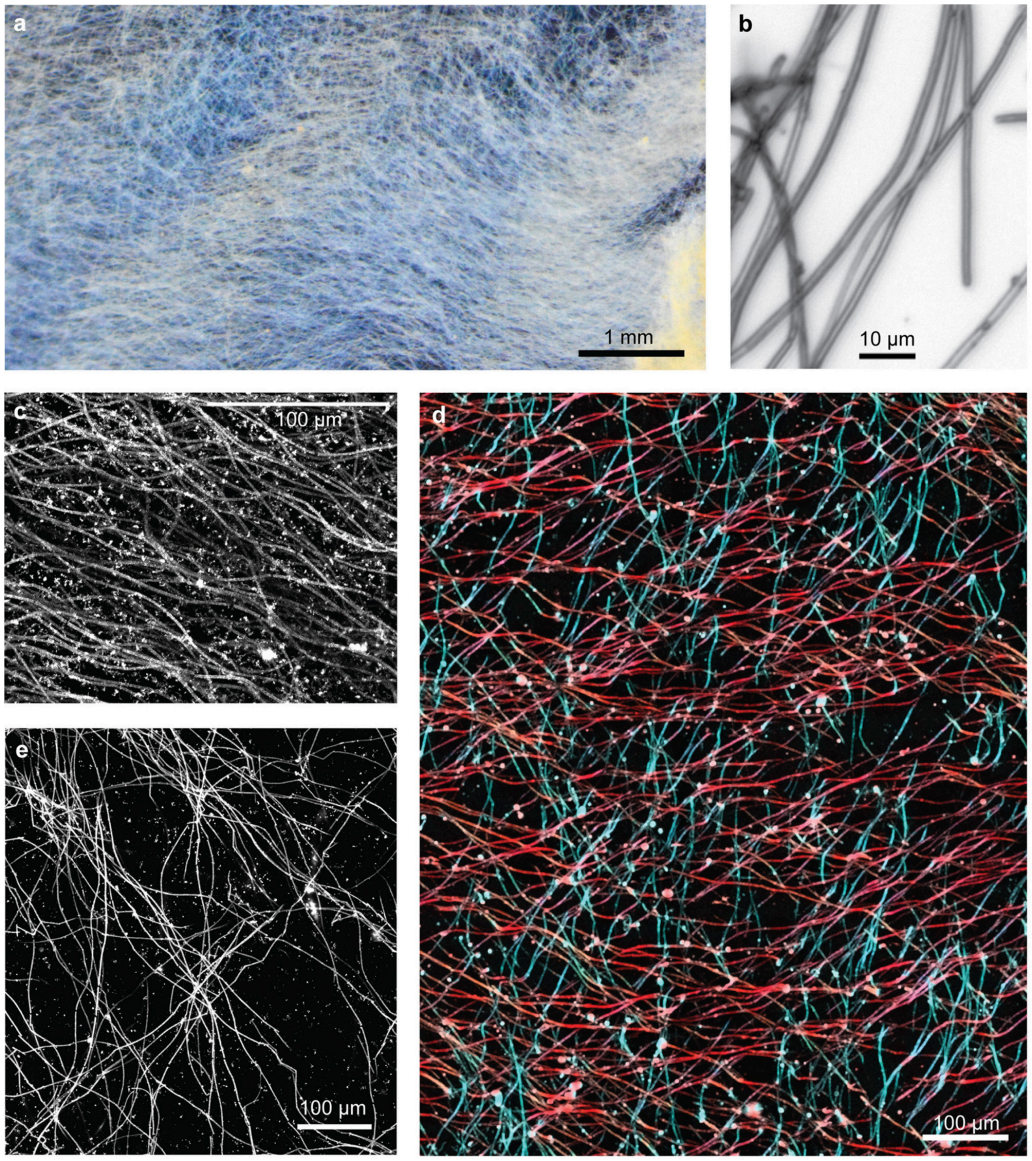
**Loihi sheath-rich veil mat**. **(A)** Stereoscope image of embedded mat section showing an overall horizontal preferred orientation. **(B)** Light micrograph showing tubular sheaths. **(C–E)** Confocal images: **(C)** sheaths in a single orientation, **(D)** sheaths in two orientations, colored in red and blue, **(E)** bundles of sheaths in multiple orientations result in web-like structure.

As expected, the veils are composed primarily of sheaths of quite uniform width (Figure 6). In some areas, sheaths were parallel or subparallel (Figure 6C), while in other areas, it appeared there were two or more sets of sheaths interwoven, with bundles of parallel sheaths (Figures 6D,E). It is difficult to tell if sheathed cells grew contemporaneously in more than one direction, or if the woven pattern resulted from the growth of successive generations of sheath-formers that each move in a uniform direction. The texture is not an artifact of sampling; since the sheaths are delicate/brittle, damage typically causes a jumble of short, randomly-oriented sheaths, unlike the intact sheaths with significant directionality shown here (Figure 6D). In fact, we rarely observed sheath ends; instead, sheaths can often be traced entirely across the length of the sample. Sheaths are observed changing direction (i.e., turning), and there was not any significant thinning or thickening of sheath mineralization; both of these features suggest that sheathed communities experience more stable environmental conditions than the stalk-forming mats.

### Colonization of marine mats by other Fe-mineralized morphologies

**Figure 7:**
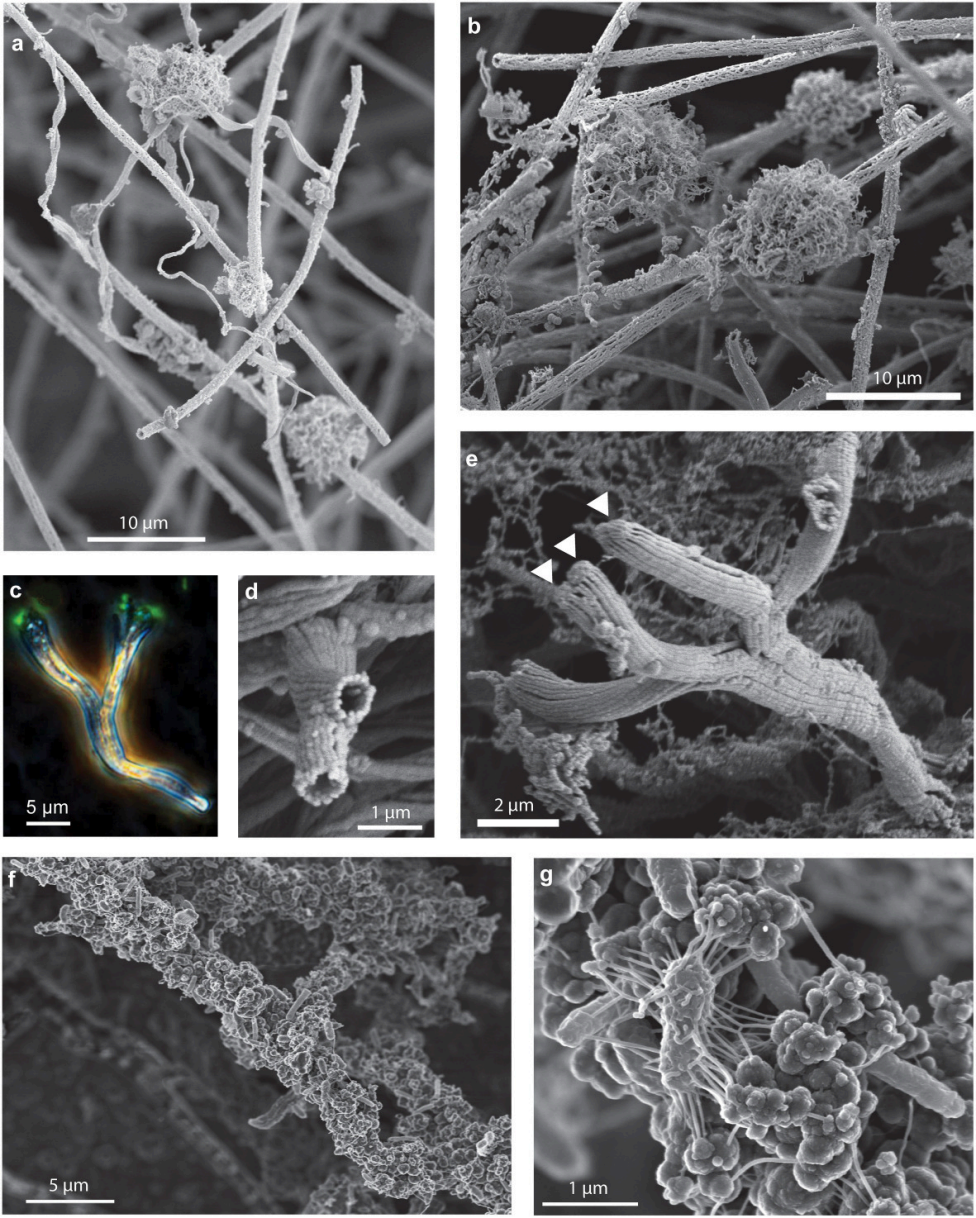
**Secondary colonizers include distinct Fe oxyhydroxide morphologies that colonize stalks and sheaths**. SEM images of **(A)** stalks and fibrillar nests colonizing sheaths and **(B)** fibrillar nests on sheaths. **(C–E)** Branching tubes: **(C)** phase contrast/fluorescence image showing branching tubes with cells (arrows) on the end. **(D)** SEM image of end-view of branching tubes missing cells, **(E)** SEM image of branching tubes with cells (arrows) on the end, **(F,G)** filaments encrusted with amorphous oxides, cells, and EPS.

Although a single morphology, stalk or sheath, comprised the primary framework of either curds or veils, both mat types included other morphologies. Because these morphologies are attached to stalks or sheaths, these likely represent secondary colonizers, and therefore can reveal insight into different ecological niches within a mat. For instance, some stalks are found colonizing sheath-rich mats (Figure 7A); however, we did not observe sheaths in the curd-mats. In all mats, stalks or sheaths were colonized by spherical structures; on closer examination by SEM, these structures are fibrillar, resembling rounded nests (Figures 7A,B). This is similar to *Siderocapsa*, a group of freshwater microbes morphologically defined by their Fe-mineralized capsules (Hanert, 2006). Like *Siderocapsa*, it is likely that a cell was at the center of the nests excreting Fe oxyhydroxide fibrils.

Another structure that has commonly been observed in marine iron mats are short (~5–50 μm) Y-shaped tubular filaments (Emerson and Moyer, 2010; Breier et al., 2012; Moeller et al., 2014; Peng et al., 2015). In the intact mats these structures were commonly observed colonizing either the sheaths or stalks (Figure 6). The Y's are composed of a flattened hollow tube made up of multiple Fe oxide fibrils. Much like stalks, a single cell can be found at the terminal end of the tube (Figures 7C,E), and when it divides the tube bifurcates producing the Y-structure; longer structures have been observed to twist. The different distributions of nests and Y's suggests that they colonize a particular geochemical niche distinct from that of the stalk- and sheath-rich mat formers. Based on these observations, it is likely these secondary colonizers are adapted to grow at the lower O_2_ concentrations that exist deeper in the mat.

Upon aging, mats become darker colored and often exhibit thicker mineralization. In these regions of the mat, filaments are thickly coated in amorphous mineralized structures and cells (Figures 7F,G), which probably represent other phylotypes and functional groups.

### Freshwater sheath-rich mat, spruce point, maine

We sampled intact freshwater iron mats at Spruce Point, Maine, from a rock-lined ditch with water depths of 3–10 cm, and a slow flow rate on the order of 0.5 cm s^−1^. The mats had complex morphologies including dams, small channels, and puffballs (Figure 2C) similar to those described by Schieber and Glamoclija (2007). Intact mat samples were collected at the leading edge of mats that had small channels (2–5 cm wide) of water flowing between them. The edge of the mat formed perpendicular to the flow of the water and was clearly visible as a lighter, whitish edge, in contrast to the more orange mats farther back from the channel. Oxygen concentrations at the mat surfaces do not differ much from those within the mat (Table 3). An Fe(II) transect showed that Fe(II) was present both in the mat and also the surrounding water, with a depletion in Fe(II) within the top centimeter of the mat (57 μM in top cm, vs. 111 μM outside mat, 190 μM inside mat; see full profile in Supplemental Figure 7).

**Table 3:** **Oxygen profiles in Spruce Point mats**.

	**1 cm in front of mat**	**Mat surface**	**1 cm inside mat**
Closer to groundwater source	22 μM	21 μM	18 μM
Further downstream from source	58	58	57
Further downstream from source	59	58	60

**Figure 8:**
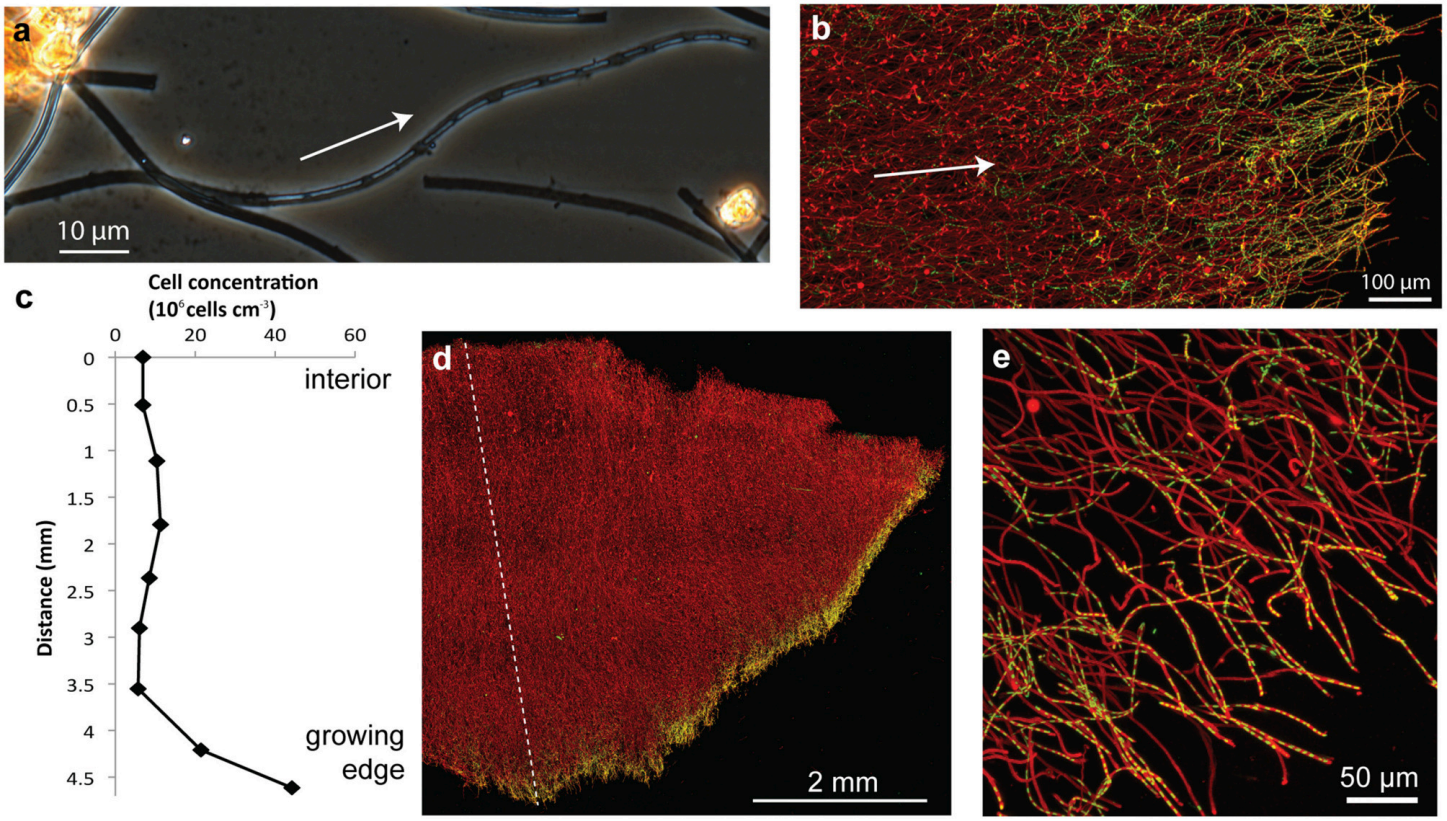
**Spruce Point mat confocal images and data showing that mat was produced by sheath-forming cells**. Arrows show inferred direction of growth. **(A)** Phase contrast light micrograph showing cells at the ends of filaments, surrounded by sheath, which is lighter (less mineralized) than empty sheaths. **(B)** Confocal image of edge of mat showing many cell filaments parallel to the direction of growth, particularly those at the mat edges; but some cells filaments behind the leading edge are non-directional. Cells (green, SYTO) and Fe oxyhydroxides (red, rhodamine-conjugated SBA lectin) **(C)** Cell concentration in transect across mat shown in **(D)**. See dashed line for cell counting transect. **(D)** Overview of intact mat showing cells concentrated at growing edge of mat. **(E)** Cells inside the ends of tubular sheaths, at the edge of the mat.

The dominant morphotype in the Spruce Point mats was hollow sheaths typical of *L. ochracea* (Figure 8). Because we were able to embed and thus preserve the mat structure within minutes after sampling, we could image the native cell distribution. Remarkably, nearly all the filaments of *L. ochracea* cells were located at the leading edge of the mat. The bulk of the mat interior was composed of empty sheaths, colonized by other bacteria (Figure 8). The terminal ends of the sheaths were visibly less mineralized than the rest of the empty sheaths (Figure 8A). The sheaths are roughly parallel to one another (Figures 8B,D,E; see Video 3 for 3D view of confocal data). Although most of the cell filaments are parallel to the overall direction of growth, a small proportion lack this directionality, which leads to some tangling that may help hold together the structure thus stabilizing the mat within stream flow.

### *Leptothrix* sheath formation and behavior

**Figure 9:**
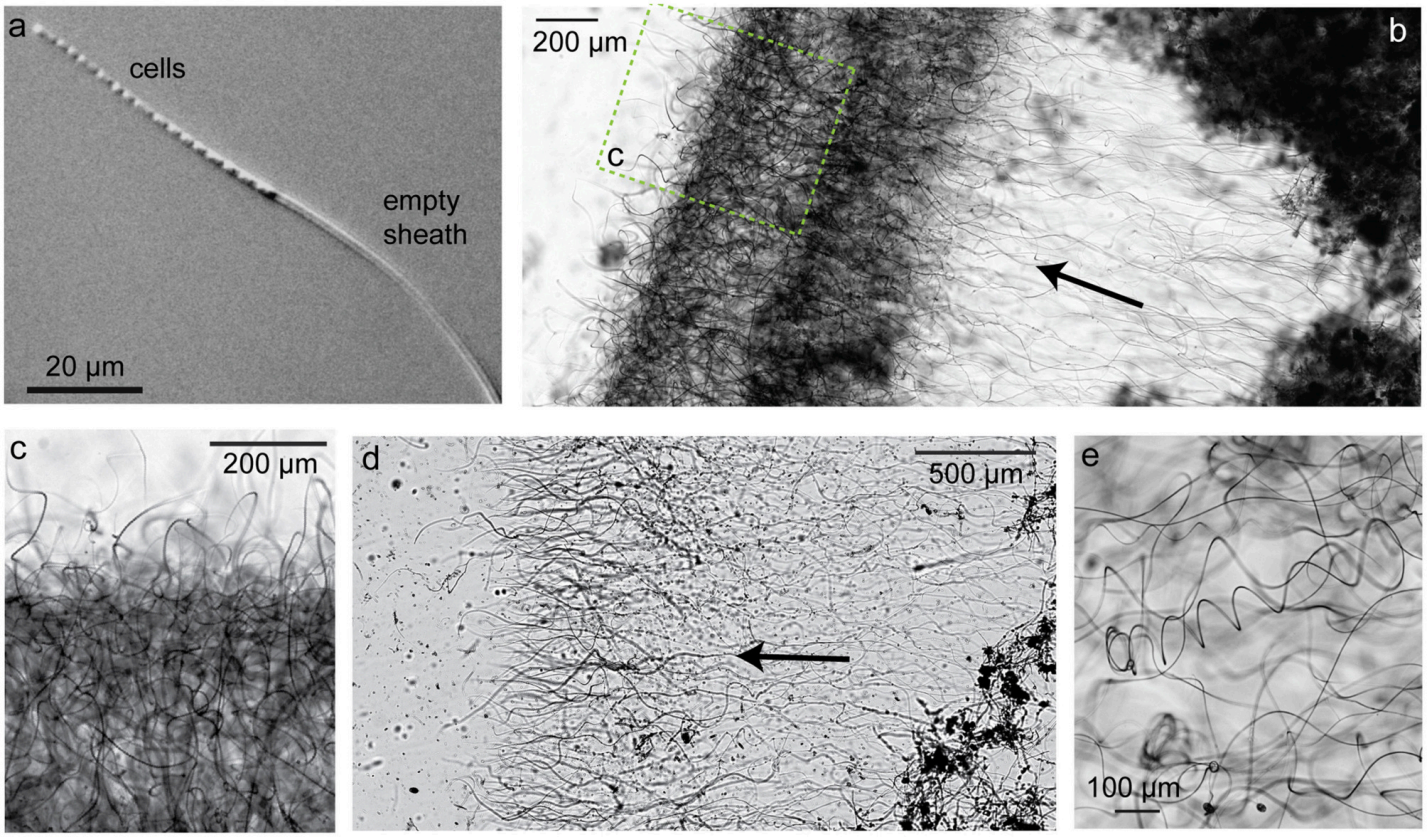
**Light micrographs of sheath-former ***Leptothrix ochracea*** enriched in a microslide growth chambers**. Arrows indicate growth direction. **(A)** Individual *Leptothrix* sheath with cells at growing end. **(B)** Directional growth (arrow) from inoculum (dark material on right) and formation of dense growth band, likely at an optimal position in redox gradient. **(C)** Enlarged detail of denser growth band. **(D)** Directional growth in another microslide. **(E)** Within the growth band, some sheaths are coiled. See also Videos 4–7.

To better understand how sheath-forming FeOB make mats, and link behavior with morphology, we used time-lapse microscopy to observe cell growth and sheath formation in opposing Fe(II)/O_2_ gradients. Using native mats as inoculum, we were able to image cells producing sheaths. Within 30–60 min of inoculation, filaments of cells were observed at the ends of sheaths (~17 cells in Figure 9A). The cells rapidly left behind empty Fe oxyhydroxide coated sheath at the trailing end (Figure 9A, Videos 4–7). This confirms that multiple cells are responsible for the formation of each sheath. Since cells continuously leave behind the sheath, it is a record of *Leptothrix* cell growth, movement, and behavior.

In densely inoculated microslides, it was possible to follow the growth and response of a population of *L. ochracea* cells to opposing gradients of Fe(II) and O_2_. Sheaths elongated away from the inoculum and FeS, toward O_2_ (Figures 9B,D and Video 7). Initially, sheaths grew in this direction rapidly (rates varied, but measured at 19 μm/min in Video 4), and the filaments were relatively straight. After some time, the sheaths formed a denser band (Figures 9B,C), and the ensheathed cell filaments showed less directionality, instead coiling to different degrees (varying radius and pitch; Figure 9E). This may be akin to the loss of directionality in certain bands of the Loihi stalk-rich mat (Figures 5E–G).

## Discussion

**Figure 10:**
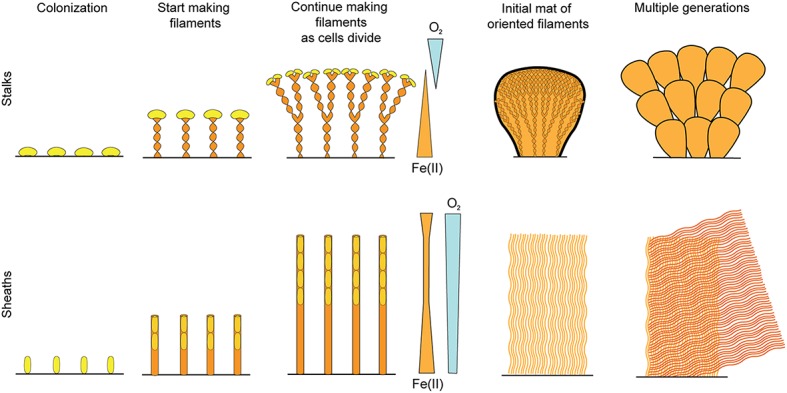
**Conceptual model of stalk- and sheath-rich mat development**.

The work presented here clearly demonstrates that FeOB produce organized microbial mats, rather than “Fe flocs,” as they are commonly referred to, since flocculation implies aggregation of cells and minerals after precipitation. Instead, filament-producing FeOB grow in coordination to produce a highly structured, albeit delicate, mat comprised of Fe oxyhydroxide biominerals that are the direct result of their lithotrophic metabolism (Figure 10). The structure of any individual mat is likely driven by the physiological and behavioral responses of specific FeOB to a combination of hydrologic dynamics and fluxes of key substrates like O_2_ and Fe(II). Obviously physiology and behavior have strong genetic components, but we observed remarkable commonalities in Fe mats produced by phylogenetically different FeOB in varied environments. Here we discuss the connections between the Fe oxidation metabolism and mat structure, focusing on how adaptations could drive the common architecture between Fe microbial mats.

### Sheath-forming organisms use biomineral filaments to attach to surfaces and remove waste, much like stalks

Our results show commonalities between Fe-mineralized stalks and sheaths that suggest they are used for similar purposes. As with stalks, *L. ochracea* sheaths are produced by cells growing at one end, leaving behind an Fe oxide mineralized filament (Figure 10). In both cases, one end of the filament is attached to a substrate, operating as a holdfast, while stalk/sheath production occurs as cells continuously produce Fe oxides. While sheaths are thought to serve as protection, e.g., from predation, dehydration, and harmful radiation (Ghiorse, 1984), such functions do not require such massive production of sheath, as is the case here. Thus, we observe that the cells use their mineral byproducts as an attachment and support structure; that is, both the twisted stalk and sheath, as well as the tubular Y structure, can be functionally considered a “stalk.”

It has been noted that most Fe sheaths are empty (Mulder and van Veen, 1963; Emerson and Revsbech, 1994); similarly, stalks are more abundant than cells, by volume. This may be explained by the low free energy available from Fe(II) oxidation, about −90 kJ mol^−1^ Fe(II), which means a significant amount of Fe is required for C fixation (~43–70 mol of Fe(II) per mol C) (Neubauer et al., 2002; Sobolev and Roden, 2004). If all of the Fe is used to make stalks or sheaths, the consequence is relatively few cells leaving behind a comparatively large mineral-based structure. This is what we observed in the Spruce Point mat (Figure 8), where the *L. ochracea* cells primarily occupy the outer fringe of the mat, which is largely composed of empty sheaths (Figure 8). In the marine mats we were not able to image cells, but in the case of the stalk-based curd mat, we can infer that cells were present at stalk ends at the mat exterior. The structure of both the stalk and sheath mats are consistent with a strategy to remove Fe(III) waste products, leave the oxides behind in the mat, and thus prevent cell mineralization/encasement.

### Both sheath- and stalk-rich Fe mats are the result of coordinated growth and motility/taxis

A common feature of the mats and the *L. ochracea* culture is the highly parallel nature of the stalks and sheaths; in addition, stalks twist in the same direction. Together, this suggests that the mat-forming cells coordinate their movement and growth, advancing as a unified front. In part, the coordinated cellular response is likely driven by external stimuli, but it is also likely there is cell-cell communication within the mat. In the alternative scenario, with neither a stimulus or communication, stalks or sheaths would be randomly oriented, and cells would not be so concentrated at the mat edge. Instead, in the mats and cultures, random filament orientation is rare and confined to specific horizons/bands as discussed below, giving further evidence that orientation represents a community response.

This unified front of cells may be a response to gradients of Fe(II) and O_2_, as seen in microslide growth experiments. The enrichments of *L. ochracea* sheaths grew in the direction of increasing O_2_ (Figure 9, Videos 4–7), much like previous observations of *M. ferrooxydans* stalk formation in microslides (Chan et al., 2011). Directional growth was also observed for *G. ferruginea*, though the direction of chemical gradients was not documented (Hanert, 1973). Once opposing Fe(II)/O_2_ gradients developed and presumably stabilized (i.e., formed an Fe(II)/O_2_ interface), the directional *L. ochracea* sheath growth ceased. At this point, we observed development of dense oxide bands that consisted of sheaths that either coiled tightly or turned perpendicular to the gradient; both strategies allow the sheath-formers to continue making the sheath while staying close to the narrow region in which they can access both Fe(II) and O_2_. This further suggests that continuous sheath formation is necessary during Fe oxidation and growth; otherwise cell growth should continue without sheath formation. Similar bands were not observed in the sheath mats from Loihi or Spruce Point, but this likely corresponds to the shallow to non-existent gradients of Fe(II) and O_2_ (see discussion in section Relationships between Filament Morphology, Niche, and Behavior). We do however, see thin bands of random orientation including some coiling in stalk mats. In this case, however, the directionality returns, raising the possibility that the mat is responding to changes in the flow and/or chemical composition of the vent fluid.

This brings us to the intriguing question of how cells move while also producing biominerals. In fact, *L. ochracea* cells appear to use biomineralization as a mechanism for motility, much like stalk-formers. In previous live culture imaging of *M. ferrooxydans*, we saw that a stalk was used to attach to substrate and then propel the cell forward (Chan et al., 2011). In the current study, we show a natural population of *L. ochracea* uses the sheath in a similar manner, but with the difference that tens of cells in a filament worked in coordination to produce a single sheath. While other organisms are known to assemble structures to propel themselves (e.g., Goldberg, 2001), a fascinating aspect of FeOB behavior is that the cells are entirely in the aqueous phase and not attached to any surface, i.e., they are not gliding along a surface or through a gel/matrix. In the case of the sheath, this raises interesting questions about how a sheathed filament of cells is able to propel itself. Cell movement would be constrained by the sheath, so are the cells extruding the sheath in such a way to direct their motion? If FeOB cells are somehow able to use production of a rigid sheath or stalk to translocate, this would represent a novel means of bacterial motility. If extrusion of biomineralized stalks and sheaths is indeed a form of motility, then the cells are cleverly using their Fe(III) waste to precisely control their position within the environment.

### Fe mat formers engineer habitats that promote Fe oxidation

Although Fe microbial mats share basic characteristics with other microbial mats and biofilms, they also have fundamental differences that are likely related to Fe oxidation. The Fe mats most closely resemble mats produced by sulfur-oxidizing bacteria, including *Arcobacter sulfidicus*, a marine S-oxidizer that produces filamentous S(0)-structures (Taylor and Wirsen, 1997; Sievert et al., 2007). Sievert et al. suggest that the filamentous S(0) is an adaptation to rapid mineralization (in this case, detoxification of high sulfide levels) and maintaining position within the oxygen/sulfide interface. However, *A. sulfidicus* is unusual amongst the S-oxidizers; others such as *Beggiatoa, Thiothrix*, and various Epsilonbacteria form streamers and other mat-like structures, much like other microbes, in which cells themselves are the primary building block of the mat (Campbell et al., 2006; Teske and Salman, 2014). Another important group of mat-building microbes, the cyanobacteria, also produce a dense network of cells bound together by extracellular polymer substances (EPS) structures, sometimes also including sheaths (e.g., Fenchel and Kühl, 2000). Degradation of EPS by other microbes is thought to result in carbonate precipitation to produce a mineralized structure (e.g., Dupraz and Visscher, 2005), but this occurs below the zone of cyanobacterial growth. In contrast to the typical mats that are dense aggregates of cells and EPS, all of the Fe mats we analyzed are primarily built from Fe oxyhydroxide filamentous biominerals with little interstitial EPS, but a vast amount of open pore space. This distinctive architecture has benefits that meet the specific challenges of chemolithotrophic Fe oxidation metabolism, most notably the need to get rid of Fe oxhydroxide waste without entombing cells or clogging flow paths through the mat.

The mat-forming FeOB thus create a framework with high surface area and permeability, which presents an opportunity for other colonizing organisms. In fact, much of the the mat interior community is likely composed of secondary colonizers. These include FeOB, as evidenced by the Fe-mineralized structures shown in Figure 7. A prime example of this is the Y-shaped FeOB that we observed colonizing the stalks and sheaths at Loihi. These fascinating organisms produce unique flattened tubular Fe-oxyhydroxide casings that have been observed previously in marine Fe mats. But for the first time here, SEM (Figure 7E) reveals a detailed juxtaposition of cells and their mineral structure, suggesting that as these FeOB grow and divide, they produce Y-shaped branching structures similar to the branching stalks. These entire structures are relatively short with total lengths that typically range from 5–50 μm, which enables them to fit into the mat structure. Likewise, other colonizing structures (e.g., nests) are also more compact than the filaments. The porous framework structure constructed by the stalks and sheaths provides plenty of room for colonizing cells, polymers, and biominerals. In this way, the stalk- and sheath-forming FeOB are keystone species and ecological engineers. They are the first to colonize the Fe-rich environment, providing a physical habitat conducive to further Fe oxidation and likely other metabolisms.

### Investigating the identities of mat-forming FeOB

Although Fe stalk- and sheath-formation is the most well-known trait of neutrophilic FeOB, and clearly critical to Fe mat formation, it appears to be a task performed by many, but not all FeOB. For instance, freshwater stalk-formers are all members of Gallionellaceae within the Betaproteobacteria, but some Gallionellaceae (e.g., *G. capsiferriformans* and *Sideroxydans lithotrophicus*) do not make stalks or any organized extracellular structure (Emerson and Moyer, 1997). Marine stalk-forming FeOB isolates are all members of the genus *Mariprofundus* within the Zetaproteobacteria (ZOTU11, as defined by McAllister et al., 2011), but new isolates of marine FeOB, including one that is nearly 97% similar by SSU rRNA to *Mariprofundus ferrooxydans*, do not form stalks either (Emerson and Barco unpublished data). These findings show that FeOB have alternative strategies for ridding themselves of Fe-oxyhydroxides (e.g., shedding smaller oxides; Kato et al., 2015; Field et al., in press), and suggest that stalk formation is a specialized task performed by specific members of the community.

Because there are not defined phylogenetic clusters of stalk- or sheath-formers, or genetic markers, we are still trying to determine the phylogenetic distribution of these organisms, to ultimately understand how these abilities evolved. Our molecular analysis of these relatively fresh, intact Loihi mats did not find members of the ZOTU 11 that includes the stalk-former *M. ferrooxydans*; instead ZOTU 6 and novel ZOTUs accounted for most of the Zetaproteobacteria. Thus it is possible that one or more of these OTUs, for which there are no cultured representatives, is a stalk former. However, as a note of caution, stalk-forming cells may be rarely observed in molecular studies since they are concentrated on the exterior of the mat (as seen in a freshwater mat; Mitsunobu et al., 2012), though we tried to include these cells by sampling the freshest mat possible. Another approach by Rassa et al. (2009) involved leaving sterile substrate near the vents for colonization. They found that ZOTU6 is a vanguard organism, consistent with our results, supporting the possibility that there are multiple groups of marine stalk-forming Zetaproteobacteria FeOB.

### Relationships between filament morphology, niche, and behavior

We observed marine stalk-formers growing at the interface of steep Fe(II) and O_2_ gradients (near vents), while sheath-formers appear to be associated with higher O_2_ concentrations in low or undetectable gradients (e.g., at the periphery of vent sites at Loihi), consistent with previous studies (Fleming et al., 2013, 2014; Krepski et al., 2013). These contrasting niches can help explain differences in filament formation and resulting morphology. Specifically, how fast cells move likely corresponds to the type of gradients they prefer. *Leptothrix* sheath formation is much faster than Zetaproteobacteria stalk formation (19 μm min^−1^, compared to 2 μm h^−1^ stalk formation rate observed for *M. ferrooxydans*), likely related to the number of cells involved. Because the sheath formers live in shallow gradients, they can likely move further than other FeOB while still maintaining their niche; this probably accounts for the lack of observable sheath ends/terminations in our centimeters-size samples. In contrast to sheaths and stalks, the branching tube “Y” structures are much shorter, which may correspond to a strategy in which they stop mineralizing and colonize elsewhere more frequently, suggesting they either have highly specific niche requirements or live in a less stable environment. Stalk twisting/coiling and sheath coiling may be related to gradient sensing and therefore chemotaxis; tighter coiling may represent efforts to minimize net motion. As we learn more about FeOB behavior, we will be able to translate morphology to environmental signals, and therefore niche. This behavior is instantly preserved as Fe oxhydroxide biominerals form, making Fe mats highly unusual in that their structure is a continuous record of FeOB metabolism.

### Prospects for interpreting Fe microfossils

If Fe mats are rapidly encased in silica or carbonate, they can be preserved long term as microfossils, and thus serve as very specific records of metabolism and niches. Indeed, there are many instances of filamentous Fe microfossils in the rock record, ranging from recent to 1.7 Ga (Little et al., 2004; Slack et al., 2007; Crosby et al., 2014), many of which appear to be intact mats. Although there is a general sense that these are biogenic, we have until recently lacked detailed characterizations of modern equivalents. Such data can help determine if these microfossils represent aerobic Fe-oxidizing bacteria, and how to interpret paleoenvironmental chemistry and setting. Recent work on *M. ferrooxydans* gave us our first detailed observations of behavior and stalk morphology (width, branching, directionality), connecting biomineral textures to Fe oxidation metabolism and oxygen preferences for a single organism (Krepski et al., 2013). Here we have extended this to natural Fe mats, which show us examples of more complex texture that can be linked to environmental and perhaps ecological dynamics. Synthesizing our culture work with observations of natural mats improves our understanding of the developmental history of the mat. This allows us to look for uniquely biological features and read the mat morphologies as a history. Because the morphologies are specific to the aerobic Fe oxidation metabolism, finding and positively identifying Fe microfossils has the potential to improve our understanding of Earth's Fe and oxygen history.

## Author contributions

CC conceived of the study and both CC and DE developed and directed the research. All authors participated in sampling, and conducted analyses, as follows: Light and confocal microscopy (SM, AL), SEM and molecular biology (SM), Loihi geochemistry (BG), Spruce point geochemistry (AL, DE), time lapse light microscopy (SK). CC wrote the manuscript, with major contributions from DE, and edits from all coauthors.

## Funding

This work was funded by NSF grants OCE-1155290 to CSC, OCE-1155754 to DE, NASA grant NNX12AG20G to CSC and DE, and a Delaware Space Grant Fellowship to SMM (NASA Grant NNX10AN63H).

### Conflict of interest statement

The authors declare that the research was conducted in the absence of any commercial or financial relationships that could be construed as a potential conflict of interest.

## Supplementary material

Video 1 **Sampling stalk-rich curd-type mat at Loihi seamount**.

Video 2 **Sampling sheath-rich veil-type mats at Loihi seamount**.

Video 3 **3D rendering of confocal imaging of Spruce Point sheath-rich mat, showing directionality of sheaths**.

Video 4 **Time lapse light micrograph series of sheath formation by multiple cells**. Inoculum from Fe seep in Rittenhouse Park, Delaware.

Video 5 and 6 **Time lapse light micrograph series of a sheaths being formed by cells**. Inoculum from Lakeside Drive, Maine Fe mat.

Video 7 **Time lapse light micrograph series of sheath formation, demonstrating growth towards higher oxygen concentration (left side of imaged area)**. Inoculum from Fe seep in White Clay Creek, Delaware.
