# Supplementary material for: The Architecture of Iron Microbial Mats Reflects the Adaptation of Chemolithotrophic Iron Oxidation in Freshwater and Marine Environments
Source: Front Microbiol. 2016 Jun 1;7:796. doi: 10.3389/fmicb.2016.00796 (PMC4888753; doi:10.3389/fmicb.2016.00796)
Supplement: Supplementary file 8 [file Image1.PDF]

Supplementary Material

The architecture of iron microbial mats reflects the adaptation of chemolithotrophic iron-oxidation in freshwater and marine environments

Clara S. Chan[1*], Sean M. McAllister[1], Anna H. Leavitt[2], Brian T. Glazer[3], Sean T. Krepski, and David Emerson[2]

1 University of Delaware, 2 Bigelow Laboratory for Ocean Sciences, 3 University of Hawaii
*corresponding author, cschan@udel.edu

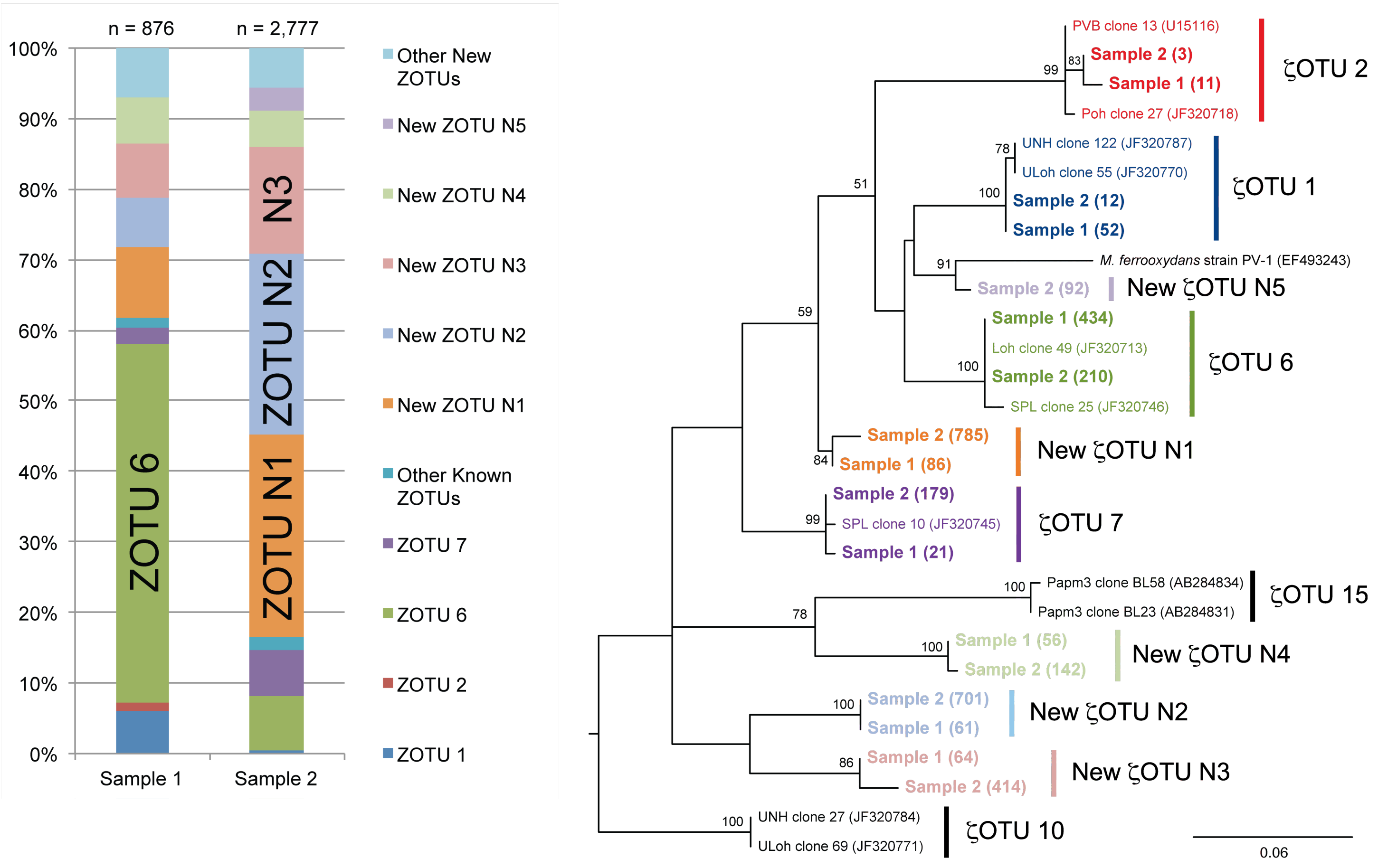

Supplemental Figure 1. (Left) Bar chart showing dominant Zetaproteobacteria OTUs in the Loihi intact curd (stalk-rich) mat samples (top four are ZOTU 6, and new ZOTUs N1, N2, and N3), which are potential mat formers. (Right) Tree showing phylogenetic placement of the dominant ZOTUs from the intact mat samples. Samples were collected from Loihi Seamount: Sample 1 = J2-675-scoop8B, collected from Marker 38; Sample 2 = J2-675-scoop6B from Marker 34. Locations are described in Glazer and Rouxel (2009).

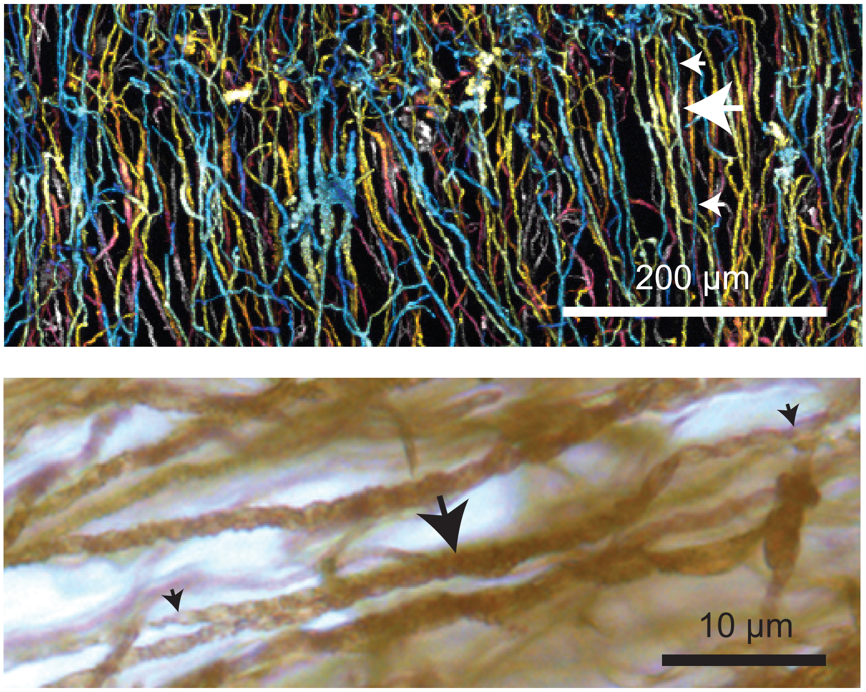

Supplementary Figure 2. Loihi curd mat stalks that thicken (large arrow), losing fine detail, and then thin (small arrow) due to temporary increase in abiotic mineralization. In culture, stalks have only been observed to thicken but not thin (Chan et al., 2011), so this is further evidence that stalks can accumulate variable abiotic mineralization, and therefore record the mineralizing potential and geochemistry of a dynamic hydrothermal environment.

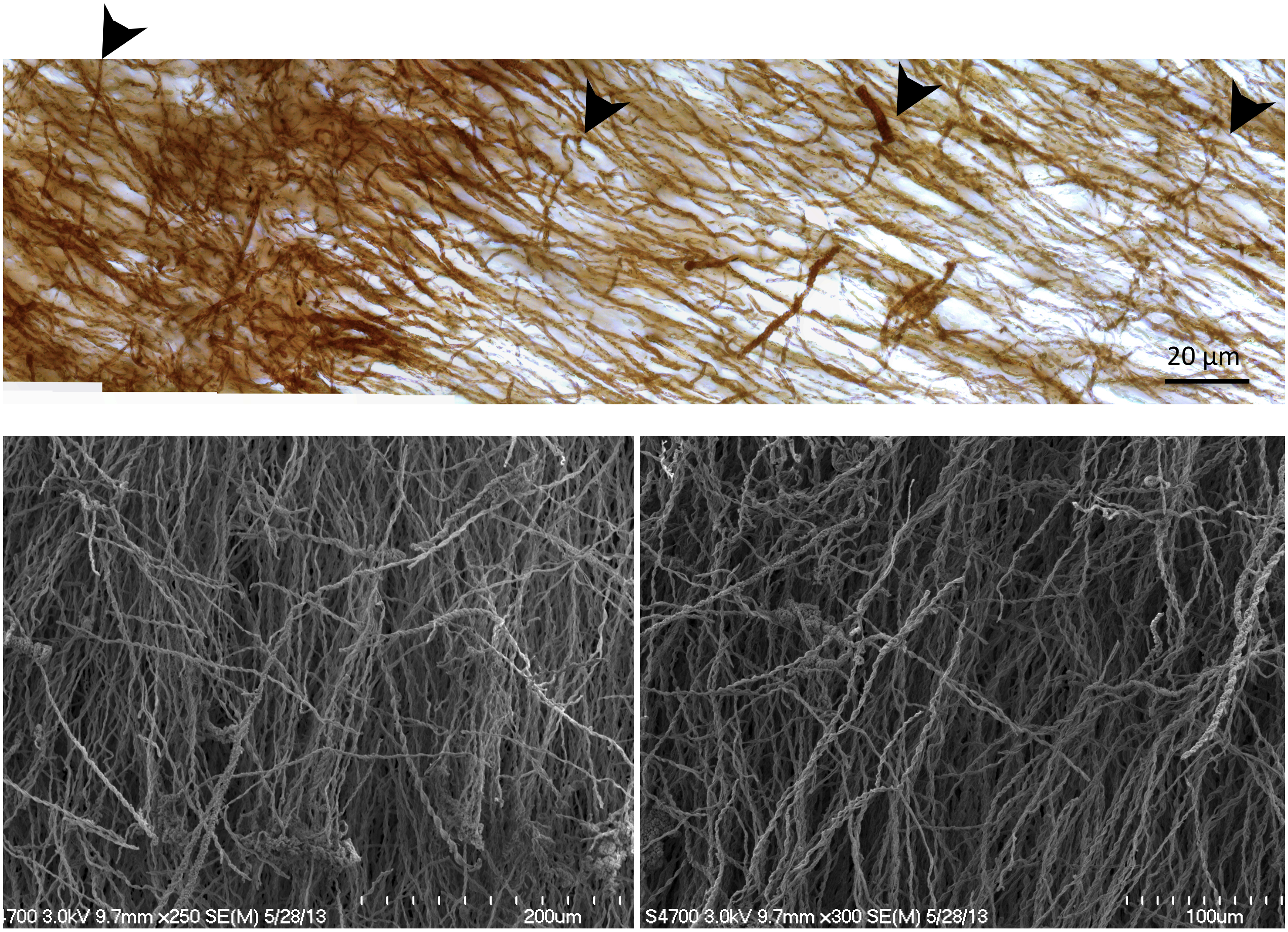

Supplemental Figure 3. Loihi curd mat images. (Top) Light micrograph of embedded section. (Bottom) SEM images of dissected mat. In highly directional areas of marine stalk-rich mats, a small proportion of stalks are orthogonal to the rest of the stalks (arrows).

Strong directionality

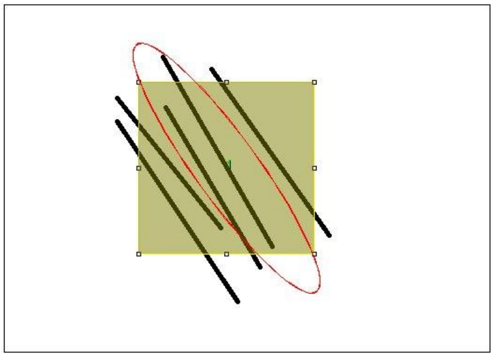

Lines:
Orientation: -53.97
Coherency: 0.666

No directionality

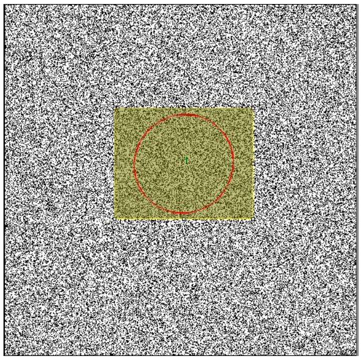

Dots:
Orientation: 43.06
Coherency: 0.015

The tan box is the the selected ROI and the red elipse shows the dominant direction

Supplemental Figure 4. Directionality analysis and metrics. Directionality was measured using the OrientationJ plugin in ImageJ, which uses stucture tensor to characterize the predominant direction of the features in a specified region of interest (orientation) and the degree to which those directions are coherent. A coherency value of 1 indicates one dominant orientation, and a value of 0 indicates complete random orientation.

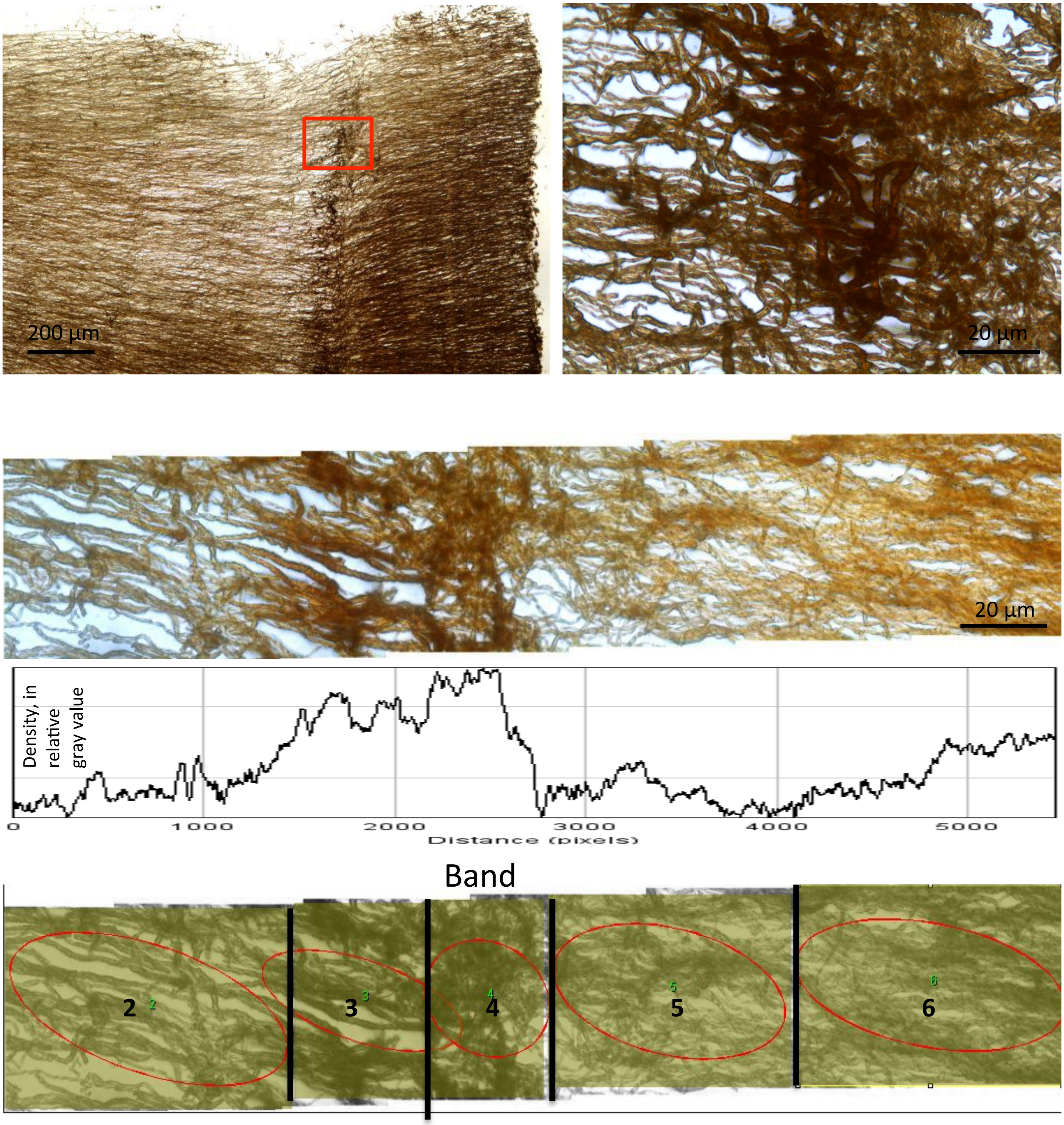

Supplemental Figure 5. Directionality analysis of a marine curd mat. Filament direction is a record of past cell location and therefore behavior. Overall mat fabrics appear to be based on highly parallel filaments, but here we highlight a band that represents a horizon in which stalks lose some of this directionality. ImageJ Orientation J results are shown in the table, corresponding to ROIs indicated above. These show loss of coherency within the band: around 0.4 before and after band, but only 0.08 within band (ROI 4). This synchonized loss of directionality, coincident with heavier encrustation indicates a temporary change in growth conditions.

ROI	Orientation	Coherency
2	-19.08	0.395
3	-18.35	0.444
4	-33.44	0.080
5	-14.23	0.308
6	-11.05	0.381

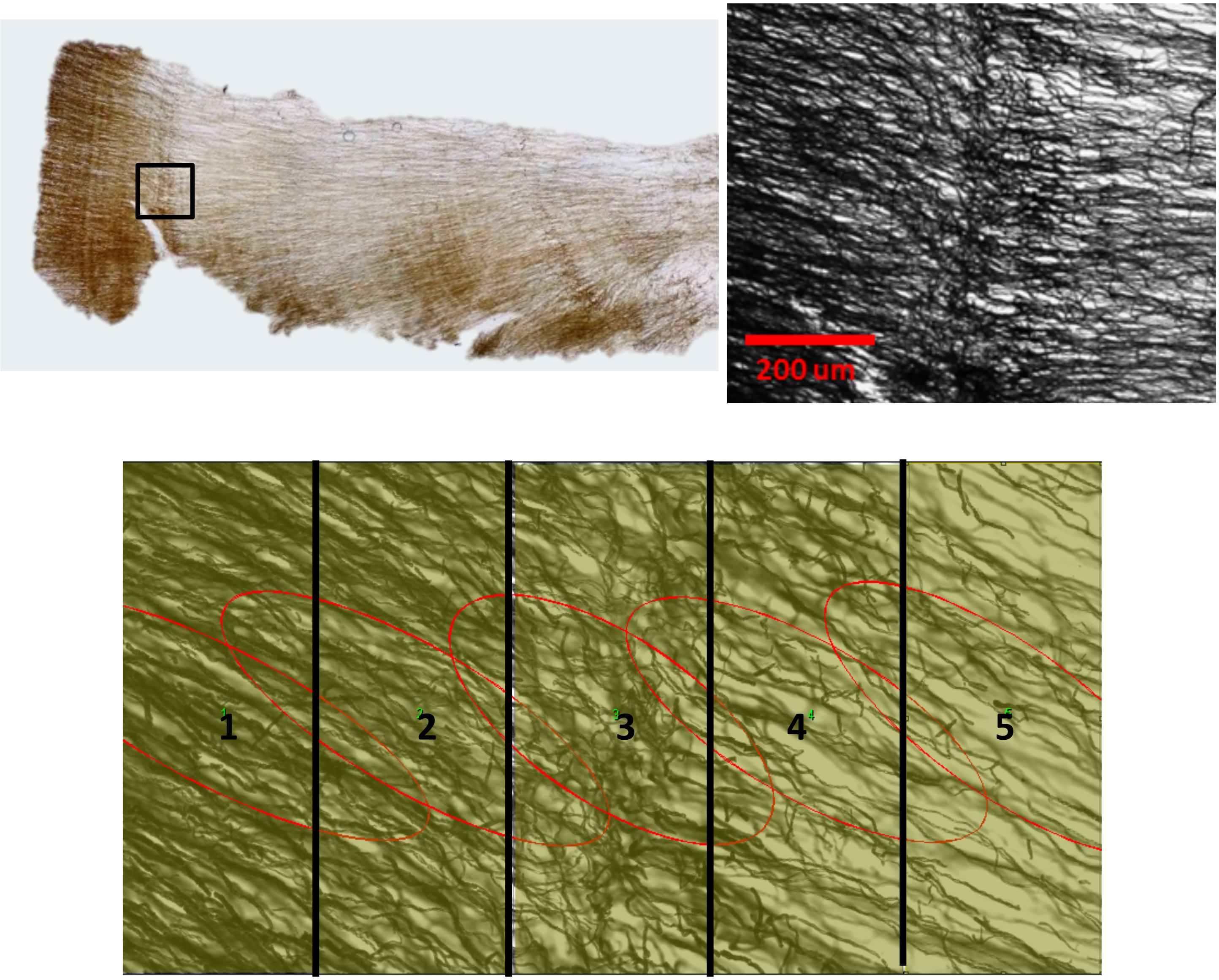

Supplemental Figure 6. Directionality analysis of a marine curd mat. Similar to Supp. Fig. 4, these light micrographs and OrientationJ analyses show loss of coherency within the band: around 0.6 before and after band, but only 0.4 within band (ROI 3).

ROI	Orientation	Coherency
1	-26.84	0.601
2	-29.77	0.557
3	-34.26	0.398
4	-29.84	0.508
5	-32.37	0.519

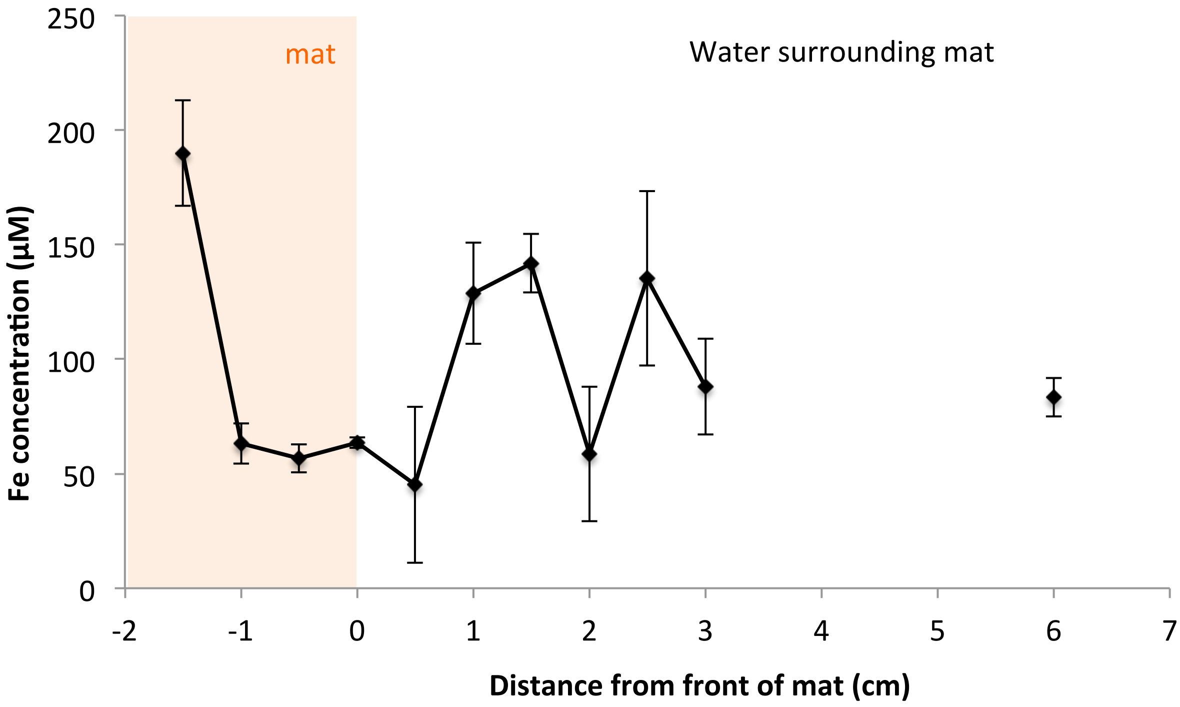

Supplemental Figure 7. Fe(II) profile across the interface of the Spruce Point freshwater mat. Values are the average of 3 measurements and error bars represent 1 standard deviation.
